# The Role of Skeletal Muscle Mitochondria in NLRP3 Inflammasome Signaling

**DOI:** 10.3390/biom16081218

**Published:** 2026-08-20

**Authors:** Jada Sangha, David A. Hood

**Affiliations:** Muscle Health Research Centre, School of Kinesiology and Health Science, York University, Toronto, ON M3J 1P3, Canada; jada20@yorku.ca

**Keywords:** NLRP3 inflammasome, skeletal muscle, mitochondria, exercise, muscle atrophy, mtROS, inflammation

## Abstract

Skeletal muscle mitochondria possess the ability to autoregulate their health and functioning by the orchestration of mitochondrial quality control (MQC) pathways. This plasticity allows them to adapt to various stimuli, such as exercise. However, under pathological conditions, mitochondria can become dysfunctional, generating damage-associated molecular patterns (DAMPs), such as reactive oxygen species (ROS) and oxidized mitochondrial DNA (mtDNA). These DAMPs can launch an innate immune response, with consequences of widespread inflammation and atrophy. Integral to this is the NLRP3 inflammasome complex. Activation of the NLRP3 inflammasome results in maturation of caspase-1, which processes pro-inflammatory cytokines IL-1β and IL-18, as well as GSDMD. Consequently, the pore-forming GSDMD-N fragment induces pyroptosis, releasing mature IL-1β and IL-18. Exercise training is widely accepted as a potent mechanism to promote skeletal muscle health, particularly by remodeling the mitochondrial network and reducing the production of DAMPs. It has also been shown promote an anti-inflammatory milieu with the release of various myokines. Indeed, the potential of exercise to mitigate NLRP3 inflammasome-mediated inflammation and atrophy is promising. This review will examine the mechanisms underpinning inflammasome priming and activation, as well the effects of exercise, with an emphasis on the skeletal muscle.

## 1. Introduction and Scope

Skeletal muscle not only makes up approximately 40% of body mass, but it is critical for performing day-to-day locomotor tasks, maintaining posture, and contributing to whole-body metabolism [1]. As such, the maintenance of muscle mass and health dictates quality of life. Skeletal muscle mitochondria are critical organelles shaping muscle health based on their environmental cues in a dynamic and plastic manner [2]. Therefore, understanding skeletal muscle mitochondria and their roles in various signaling pathways is paramount.

The scope of this review is to examine the innate immune signaling pathway central to the NLRP3 inflammasome, with an emphasis on its molecular mechanisms and, where possible, the implications and relations to skeletal muscle. Additionally, due to the influential role of mitochondria on skeletal muscle homeostasis, this review will focus on the influence of mitochondria on NLRP3 inflammasome signaling. The potential for exercise training as a promising therapeutic will also be discussed. The current literature surrounding the NLRP3 inflammasome has yet to thoroughly explore skeletal muscle. Therefore, understanding this signaling pathway within muscle, the contributions from mitochondria, and the benefits of exercise training requires further elucidation.

## 2. Muscle Immune Response Overview

The innate immune response is the body’s first line of defense against harmful stimuli, characterized by its fast-acting and non-specific nature. Conserved molecules from invading microbes, such as viral DNA, and bacterial cell membrane components (lipopolysaccharide, LPS) known as pathogen-associated molecular patterns (PAMPs), as well as endogenously synthesized damage-associated molecular patterns (DAMPs) released under cellular damage or death, are recognized by pattern recognition receptors (PRRs). PRRs are found on many cells like macrophages and neutrophils. Additional immune cells get recruited to the site of infection by cytokine signaling. Ultimately, these immune cells work to clear damage and shift signaling from a pro-inflammatory to an anti-inflammatory state. Alternatively, antigen presentation initiates the adaptive immune response, a slower and more specific response, if required. This is supported by the roles of cytokines, immune checkpoint molecular pairs, and metabolic sensors. The adaptive response relies on B cells and T cells. This evolutionarily conserved system is critical for survival, removing harmful invaders, clearing damage, and promoting tissue regeneration [3].

Skeletal muscle has emerged as an immune organ with involvement in both the innate and adaptive response. In addition to muscle-residing macrophages and infiltrating immune cells [4,5], muscle cells can coordinate their own immune signaling. Muscle cells independently sense invaders through the expression of multiple PRRs, including toll-like receptors (TLRs) and nod-like receptors (NLRs) [6,7]. These receptors can be both membrane-bound and intracellular, indicating that muscle is responsive to both PAMPs and DAMPs. These PAMPs and DAMPs induce downstream signaling, most notably activating NF-κB, a transcription factor responsible for regulating a multitude of genes, including pro-inflammatory cytokines [8,9,10,11]. These cytokines may be secreted, propagating an immune response by activating membrane-bound cytokine receptors [12] and recruiting immune cells to infiltrate the muscle. Apart from this autocrine signaling, cytokines also act in an endocrine fashion [13], expanding this inflammatory milieu systemically. IL-6 is the one of the most extensively studied cytokines secreted by contracting muscle, and this contributes largely to IL-6 serum levels [14]. IL-6 signaling promotes glucose metabolism, lipolysis, and myogenesis, amongst other functions [15,16,17]. In a context-dependent manner, IL-6 exerts both anti-inflammatory and pro-inflammatory effects [18,19]. Additionally, skeletal muscle cells function as non-professional antigen-presenting cells (APCs) due to their expression of MHC-I and -II, as well as cell adhesion molecules in conditions of inflammation [6,20,21], thus modulating T cell activation. Over time, appreciation for the ability of skeletal muscle cells to interact with immune cells, sense PAMPs and DAMPs, and launch their own inflammatory response has grown; consequently, the organ is regarded as a potent immune signaling hub. This is critical for muscle regeneration and repair after injury, as well as myogenesis; however, pathologies ensue when the highly coordinated system becomes dysregulated.

## 3. Skeletal Muscle Mitochondria

### 3.1. Overview

Within muscle, mitochondria exist in two distinctive pools, termed subsarcolemmal (SS) and intermyofibrillar (IMF) mitochondria [22,23]. SS mitochondria are located below the sarcolemma, whereas IMF mitochondria form elongated, fused networks between myofibrils [22,23]. The reticular nature of the mitochondrial network allows for optimized energy production [24].

Mitochondria are constantly subjected to quality control processes, which allow for the maintenance and remodeling of this reticular pool [25]. This includes mitochondrial biogenesis and mitophagy, which regulate de novo synthesis and selective degradation, respectively [25]. Additionally, opposing fission and fusion processes work in balance to both fragment and connect mitochondria [25]. Adaptations responsive to various energy demands, including exercise and disuse, occur by altering the drive towards these aforementioned mitochondrial quality control (MQC) processes, reshaping the organelle network and, consequently, influencing muscle quality [26,27,28].

The most prominent responsibility of mitochondria is the generation of ATP via oxidative phosphorylation. Concentrated within the cristae are electron transport chains (ETCs) consisting of protein complexes used to shuttle electrons and ultimately produce ATP. Under physiological conditions, the ETCs work effectively to supply the cell with energy, while emitting a minimal level of reactive oxygen species (ROS) as byproducts. These ROS act as signaling molecules, and are critical for cellular homeostasis [29]. However, under pathological conditions, ROS production is elevated, leading to oxidative stress [30].

### 3.2. Mitochondria and Immune Signaling

A major origin of immune signaling with in muscle are mitochondria. These metabolic organelles are highly plastic, though under pathological conditions they become dysfunctional. Dysfunctional mitochondria produce DAMPs, including ROS and oxidized mitochondrial DNA (ox-mtDNA), and they release cytochrome c and cardiolipin [31,32,33]. In fact, mitochondria are one of the main sources of ROS production [34]. Additionally, due to the proximity of mtDNA to ETCs and therefore ROS, these unstable molecules are able to effectively oxidize mtDNA [35,36]. The lack of protective histones also makes mtDNA highly susceptible to oxidation [37,38]. These damaged mitochondria propagate retrograde signaling to the nucleus, whereby DAMPs initiate a nuclear-encoded inflammatory gene program of upregulation [39,40]. Despite the intentions for homeostatic maintenance, this has the capability to be maladaptive with chronic signaling and potentiate the inflammatory milieu, attenuating mitochondrial function even further. For example, IL-6, IL-1β, and TNF-α have been associated with reduced mitochondrial function, demonstrating the vulnerability of mitochondria to their surrounding pro-inflammatory environment [41,42,43]. This positions mitochondria as critical players in immune signaling and reveals a bifunctional relationship between mitochondria and inflammation.

### 3.3. Exercise and Disuse

Exercise training is a potent modulator of mitochondrial health. It acts most notably by initiating several signaling cascades converging on PGC-1α activation and driving mitochondrial biogenesis via transcriptional mechanisms [25]. Moreover, fusion is elevated compared to fission, promoting a more elongated morphology [44]. These changes favor an increased content and function of mitochondria. Conversely, disuse leads to a more fragmented morphology, resulting from greater fission and impaired mitophagy [45]. Biochemically, these mitochondria exhibit an increased production of ROS and decreased respiration [30]. Elevated ROS drives the opening of the mitochondrial permeability transition pore (mPTP), which leads to swelling and apoptotic signaling via the release of pro-apoptotic proteins [45]. In addition, oxidative stress propagates an inflammatory response [46]. These factors ultimately culminate in muscle atrophy. Therefore, exercise training serves to mitigate this maladaptive response by inducing mitochondrial remodeling.

## 4. The NLRP3 Inflammasome

### 4.1. Function and Overview

The NLRP3 inflammasome is a multimeric protein complex, and is among the most well-studied inflammasomes. It falls within the class of nod-like receptors (NLRs), which comprise cytosolic PRRs [47]. Acting as both a sensor and an effector, it is involved in propagating the innate immune response, which culminates in inflammatory cell death, termed pyroptosis [47]. It was first discovered in 2001 by Jurg Tschopp [48]. Gain of function mutations in NLRP3 result in the umbrella cryopyrin-associated periodic syndrome (CAPS), which encompasses familial cold autoinflammatory syndrome (FCAS), Muckle–Wells syndrome, and neonatal onset multisystem inflammatory disease (NOMID) [49,50,51,52]. In addition to these autoimmune diseases, type 2 diabetes, Parkinson’s Disease, rheumatoid arthritis, and aging are just some conditions that have been associated with NLRP3 mutations [53,54,55,56]. Its ability to surveille a number of diverse PAMPs and DAMPs, as well as its general disruptions in cellular homeostasis and implications in various pathologies, make the NLRP3 inflammasome an intriguing target to study. While much of the relevant literature has been focused on immune cells, skeletal muscle cells have indeed been shown to express NLRP3 [57,58,59], posing a risk for various pathologies, and therefore require characterization and investigation.

### 4.2. NLRP3 Inflammasome Structure

**Figure 1:**
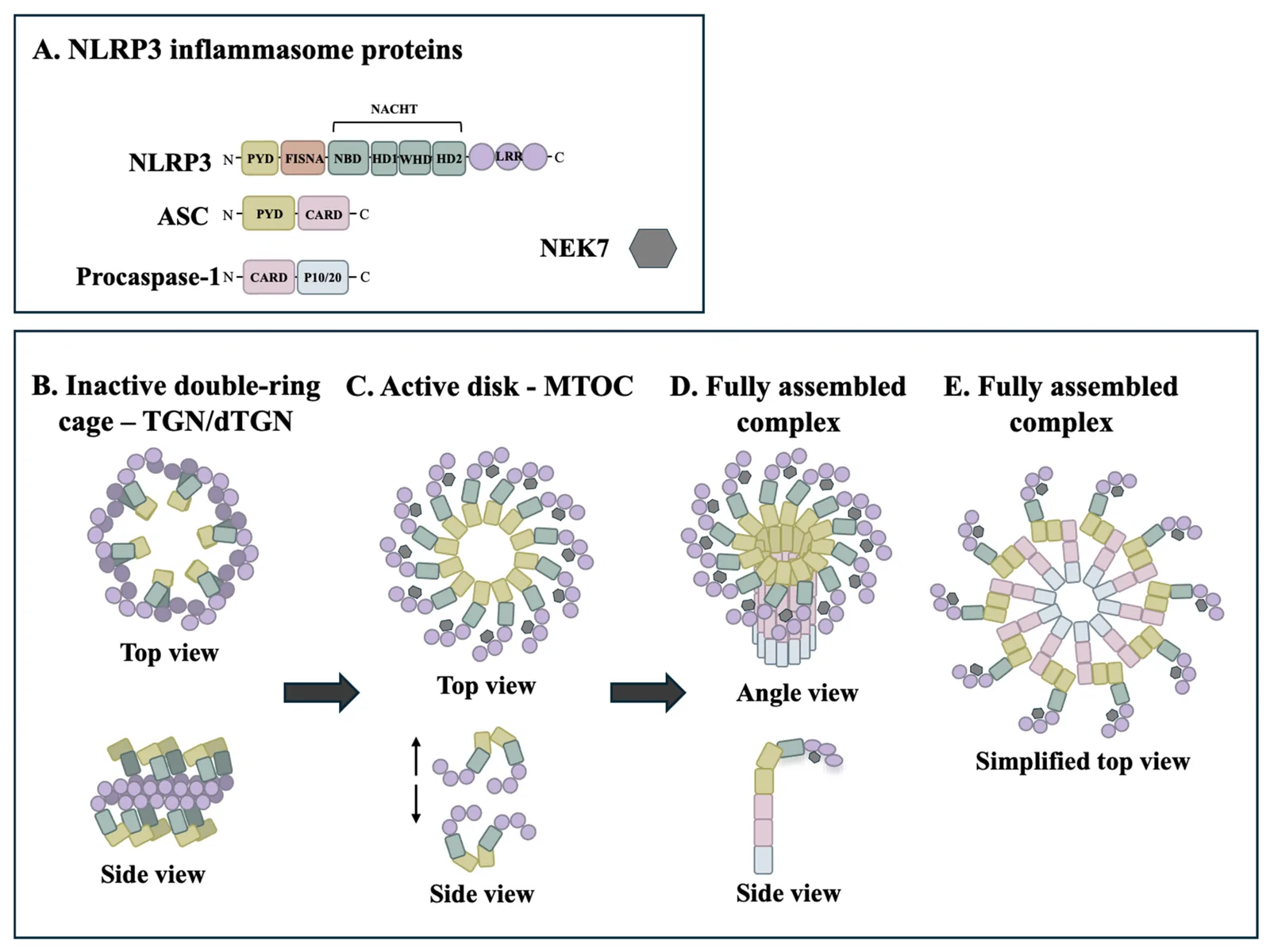
NLRP3 inflammasome structures. (**A**) Illustration of the three proteins, NLRP3, ASC, and procaspase-1, involved in forming the NLRP3 inflammasome complex. NLRP3 contains an N-terminal pyrin domain (PYD); fish-specific NACHT-associated domain (FISNA); NACHT domain and its subdomains, including the nucleotide-binding domain (NBD), helical domain 1 (HD1), winged helix domain (WHD), and helical domain 2 (HD2); and the C-terminal leucine rich-repeat (LRR) domain. The ASC protein comprises an N-terminal PYD and a C-terminal caspase recruitment domain (CARD). Procaspase-1 has an N-terminal CARD with a large catalytic subunit (p20) and a small catalytic subunit (p10) on the C-terminal. Additionally, NIMA-related kinase 7 (NEK7) interacts with NLRP3 during inflammasome activation. (**B**–**E**) The sequential NLRP3 structures illustrated from different view aspects during priming and activation. (**B**) Inactive double-ring cage structure formed by approximately 10–16 NLRP3 proteins on the trans-Golgi network (TGN) and the dispersed TGN (dTGN). (**C**) The active disk structure, promoted by the addition of NEK7 binding, located on the microtubule-organizing structure. This conformation recruits ASC, leading to inflammasome assembly. (**D**) The fully assembled complex, including NLRP3, ASC, and procaspase-1. (**E**) A simplified illustration of the fully assembled NLRP3 inflammasome complex.

The NLRP3 inflammasome is an intricate and dynamic structure. It comprises the NLR family pyrin domain containing 3 (NLRP3) protein, apoptosis-associated speck-like protein containing a CARD (ASC), and procaspase-1 (Figure 1A). NLRP3 contains a C-terminal leucine-rich repeat (LRR) domain, and an N-terminal pyrin domain (PYD), which flank the FISNA and NACHT domains. Within the NACHT domain are several subdomains, including the nucleotide-binding domain (NBD), helical domain 1 (HD1), winged helix domain (WHD), and helical domain 2 (HD2) [60,61]. Functionally, the NBD possesses ATPase activity and binds ADP in the autoinhibited state, while ATP is associated with the active conformation [62]. The HD1 and WHD undergo conformational changes associated with activation [63]. Notably, the LRR domain interacts with NEK7 [64]. The PYD is responsible for PYD-PYD homotypic interactions with ASC, allowing prion-like ASC speck formation to act as a scaffold for procaspase-1 binding [65,66,67]. ASC also takes part in CARD-CARD interactions, with the zymogen, procaspase-1, on its N-terminal [68]. At the C-terminal is the small catalytic subunit domain, p10, while in the middle is the large catalytic domain, p20 [69].

## 5. Signal 1: Priming

### 5.1. Regulation Overview

Owing to its ability to mount an innate immune response with deleterious consequences, the NLRP3 inflammasome complex is tightly regulated, requiring two signals and assuming multiple structural conformations as additive security measures. In regard to the activation mechanism, both priming and activation signals are necessary in most cases for full activation [70,71]. Classically, the priming signal allows for transcriptional upregulation of the machinery needed for downstream complex assembly [70]. More recently, early-phase or rapid priming involving post-translational modifications (PTMs) have also been described [72]. The activation signal confers full inflammasome assembly and activation [47,73].

### 5.2. Priming Signaling Pathway

**Figure 2:**
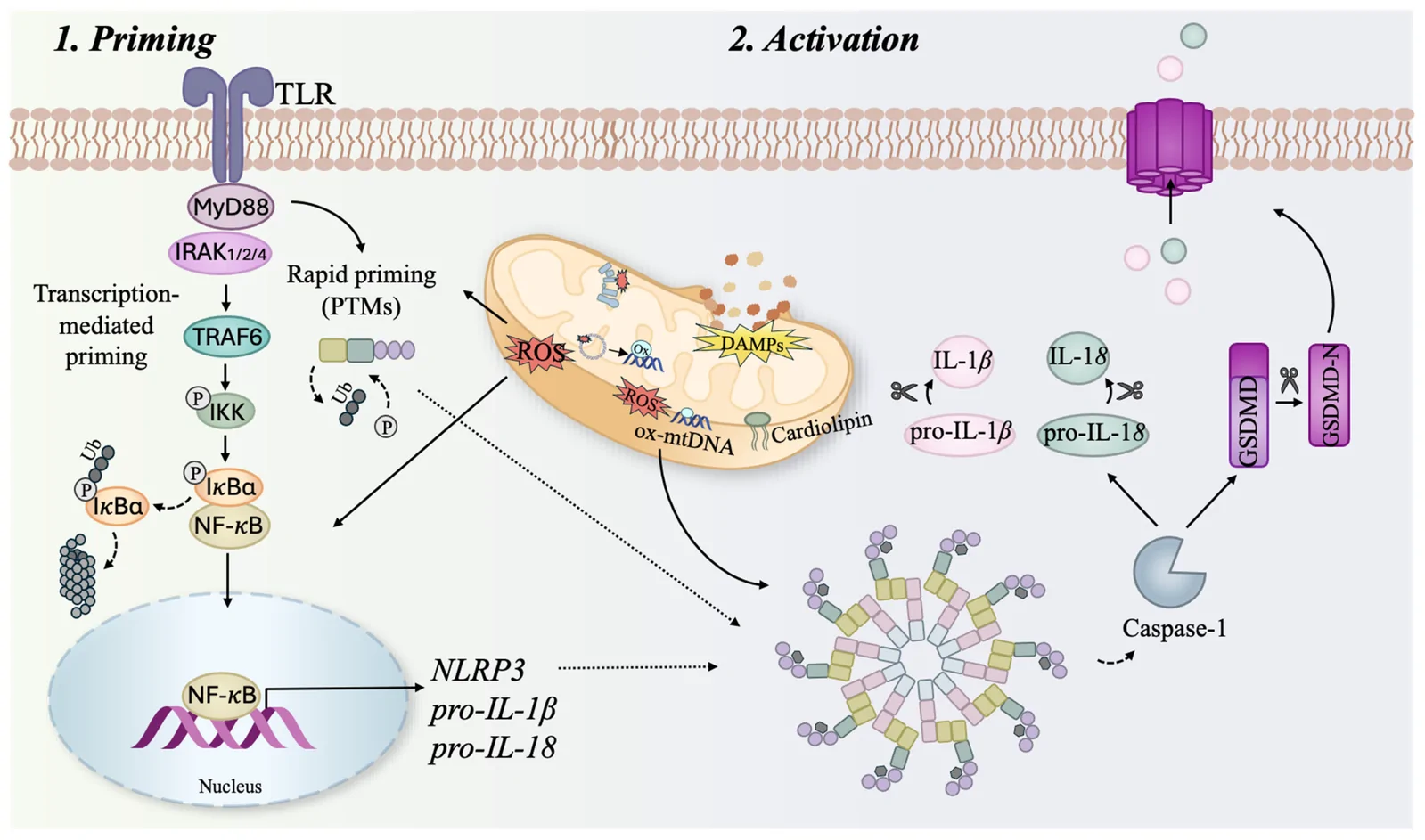
NLRP3 inflammasome signaling pathway. The prerequisite priming signal occurs via activation of various PRRs, such as TLRs. Initially, priming is mediated by various PTMs, including but not limited to deubiquitination and phosphorylation of NLRP3, while longer durations allow for the transcription-dependent response, whereby the Myeloid differentiation primary response 88 (MyD88) acts as an adaptor upon activation of TLR. This leads to the formation of a signaling complex, which includes IRAKs. TRAF6 is then activated, while downstream of this, IKK is phosphorylated and activated. Consequently, IκBα is phosphorylated, promoting its ubiquitination and degradation. This allows for NF-κB to translocate to the nucleus where it upregulates transcription of NLRP3, pro-IL1β, and pro-IL-18. Upon an activation signal, such as mtROS or ox-mtDNA, amongst many others, NLRP3 inflammasome oligomerization occurs, recruiting ASC and procaspase-1. This close proximity of procaspase-1 proteins leads to autocleavage, resulting in the biologically active caspase-1, which processes pro-IL-1β and pro-IL-18 into their mature forms in order to be secreted. Additionally, caspase-1 cleaves GSDMD, leaving the GSDMD-N to insert itself into the membrane and oligomerize into a pore. As a result, pro-inflammatory cytokines may be released, the cell swells, and eventually pyroptosis occurs.

Priming of the inflammasome is most often a result of TLR activation; however, it may also act through cytokine receptors, such as TNFRs [70,71]. Activation of TLR4 by lipopolysaccharide (LPS), a component of the cell wall of Gram-negative bacteria found to be elevated in sepsis [74], is most commonly used experimentally in a model of priming due to its efficacy. Upon stimulation of TLR4 and IL-1R, MyD88, an adaptor protein, mediates the activation of IRAK1/2/4 [75,76,77]. In turn, TRAF6 E3 ubiquitin ligase activity adds K63-linked ubiquitin chains, which recruit the complexes of TAK1, TAB2, and TAB3, where TAK1 phosphorylates IKK [78,79,80,81]. IKK then promotes the ubiquitination and subsequent degradation of IκBa via phosphorylation, relieving NF-κB from sequestration and allowing for nuclear translocation [82,83,84]. NF-κB may then promote the transcription of NLRP3 and IL-1β [11,85], allowing for subsequent activation (Figure 2). Indeed, the NLRP3 promoter region contains two NF-κB binding sequences that are critical for NLRP3 promoter activity [85]. The rapid priming response, which is transcriptionally independent, remains to be fully elucidated. However, in BMDMs, similar to the aforementioned pathway, the TLR-MyD88-IRAK1/4 axis was critical for the rapid activation of NLRP3 [86,87].

## 6. Signal 2: Activation

The priming signal upregulates the protein expression of NLRP3 and IL-1β as well as promotes PTMs that license an activation state of proteins involved, thereby setting the stage for future activation. Basally, NLRP3 exists as monomers and dimers in the cytosol [88]. However, upon priming and activation signals, the structure and subcellular location of NLRP3 becomes an intricate and dynamic process mediated by various PTMs, which remain to be fully understood. Priming promotes NLRP3 oligomerization to form a closed double-ring cage/barrel structure, ranging from 12 to 16 units in a mouse model or 10 units in a human model, as revealed by cryo-EM imaging [63,64,89] (Figure 1B). The responsibility for this structure is due to predominantly LRR-LRR interactions [90], as well as PYD-PYD [64,89]. PYDs remain inaccessible, tucked within the cage, rendering the NLRP3 cage autoinhibited prior to activation [64,89]. This cage-like structure appears to localize on membranes [89], like the TGN, mediated by ZDHHC1 palmitoylation [91], and ZDHHC7 palmitoylation [92] though monomers and dimers remain in the cytosol [64]. Subsequent activation signals lead to the disruption of the TGN, resulting in dispersed TGN (dTGN) vesicles in which NLRP3 may be trafficked [64,91,92,93]. Additionally, HDAC6 and MARK4 have been shown to transport NLRP3 associated with dTGNs to the MTOC, which acts as a scaffold by promoting ASC association [91,94,95,96]. Indeed, catalytically active caspase-1 was seen surrounding the inflammasome in addition to IL-1β, indicating that the MTOC is a platform for complete NLRP3 inflammasome activation and the downstream processing of IL-1β [94]. However, another study found no localization of ASC specks with the MTOC [97]. The exact subcellular location of NLRP3 during activation continues to be resolved, as studies cite the TGN, MTOC, endosomes, lysosomes, ER, and mitochondria (as described in Section 8.7) all as platforms for activation [98]. Therefore, it has been suggested that a context-dependent translocation response occurs, which depends on the type of organellar stress [98]. Interestingly, in addition to the role of transducing immune signaling, MTOC localization appears to be associated with autophagy, revealing a bifunctional role [94]. NEK7 is enriched at the MTOC [99] and has been shown to be required for NLRP3 inflammasome activation at the MTOC [91,100]. The steric clash between NEK7 and the LRR domain of NLRP3 results in the dissociation of the cage [64,73], which has been shown to form NLRP3-NEK7 dimers [90]. This disrupted cage may act as an intermediate for sequential reorganization to form the active disk like structure [73]. This active disk is formed by 10 units in a human model, in which LRR and NEK7 no longer contribute to the structure, instead FISNA and NACHT domains are responsible for interactions [73] (Figure 1C). The filamentous NLRP3-PYD structure facilitates the nucleation of ASC-PYDs, which ultimately aggregate, resulting in an ASC speck [65,73] which is a common readout of inflammasome activation. This ASC speck acts as a scaffold, recruiting and binding procaspase-1 [65] (Figure 1D). Due to its proximity, procaspase-1 undergoes autocatalytic cleavage, resulting in the p33/p10 product, which is further cleaved and liberates the p20/p10 heterotetramer, where its instability renders it inactive [65,69]. The proteolytically active caspase-1 then cleaves GSDMD, yielding the N-terminal fragment. GSDMD-N, which inserts itself into the cell membrane via interactions with lipids, oligomerizes to create a pore [101]. This pore formation leads to cellular swelling and ultimately cell death by pyroptosis [101]. Additionally, inflammatory factors including IL-1β, which is processed into its mature form by caspase-1 [65,102], propagate the inflammatory phenotype (Figure 2).

## 7. Intricacies of Priming

### 7.1. Transcriptional Priming

The necessity of transcriptional upregulation, however, has faced questioning. Murine macrophages are unable to form ASC specks with LPS or ATP alone; however, with both the priming and activation signal, inflammasome activation is successful [70]. Similarly, a priming signal initiated by either TNF-α or LPS in addition to ATP was required for caspase-1 cleavage, indicating a requirement for priming in NLRP3 inflammasome activation [71]. Additionally, administration of cycloheximide, which inhibits protein synthesis, showed inhibition of NLRP3 activation in BMDMs primed with LPS for 4 h [70], or with TNF-α for 6 h [71]. Further, this response was dependent on NF-κB signaling [70,71]. Indeed, p-NF-κB, p-IκBα, and NLRP3 elevations are observed in concert within the muscle [103].

NLRP3 overexpression in macrophages reveals that priming is a dispensable process, with evident activation in under 1 h of nigericin stimulation alone [70]. Similarly, macrophages expressing elevated levels of NLRP3 exhibit greater caspase-1 processing and at a faster rate with priming and activation, as well as with ATP alone [72], thus revealing that NLRP3 expression is a key mediator of the priming step. This provides a basis for other work showing that priming may be dispensable. Indeed, monocytes and dendritic cells, when compared to other hematopoietic cells, express NLRP3 most at high levels [104]. Human monocytes treated with a common activator—nigericin for 45 min—appear to bypass the priming step and secrete IL-18, which is constitutively expressed to levels similar to their primed counterparts [105]. Additionally, cleaved caspase-1 was also upregulated in the unprimed condition [105]. Human monocytes can also respond to a priming signal alone, specifically LPS [106], but this may not be required. The necessity for transcriptional priming remains unclear with inconsistent results in dendritic cells [106,107]. Research in skeletal muscle cells is extremely limited. However, the NLRP3 inflammasome is activated in muscle cells with LPS alone, though a 72 h treatment duration was required [58,108]. Therefore, in muscle, one extended signal may be sufficient for inflammasome activation. In summary, the necessity of priming may be NLRP3 expression-dependent [70,72]. The differential results described reflect a cell-type- and species-dependent priming response, positing NLRP3 expression as rate-limiting for inflammasome activation.

### 7.2. Transcriptionally Independent Priming

Rapid priming was first characterized in 2012 and promoted the exploration of early- and late-phase priming [72]. Rapid priming is transcriptionally independent, as shown by a lack of change in caspase-1 when BMDMs were treated with cycloheximide and LPS stimulation for 10 min and then 45 min of ATP [72]. The authors pinned the discrepancies seen in the aforementioned cycloheximide studies on the length of LPS treatment. Longer priming relies on transcriptional regulation whereas shorter durations are independent [70,72]. As such, caspase-1 cleavage is seen to precede elevations in NLRP3 protein expression at as early as 10 min of LPS priming and 45 min of ATP stimulation [72,86]. Comparatively, NLRP3 elevation did not occur until after 2 h of LPS priming and a subsequent 45 min of ATP stimulation [72]. This is supported by other groups, who also observed transcriptionally independent priming in the early phase [109,110]. This rapid priming response appears to be mediated by various post-translational modifications (PTMs), such as ubiquitination, phosphorylation, palmitoylation, and sumolyation, which effectively license the inflammasome for subsequent activation [111].

### 7.3. Post-Translational Modifications (PTMs)

Ubiquitin chains may serve dual roles in regulating the inflammasome, including the canonical signaling for proteosome-mediated degradation by the K48-linked polyubiquitin chain [112,113], as well as initiating alternative signaling pathways, by the K63-linked polyubiquitin chain [79,114]. Basally, NLRP3 appears to be in a ubiquitinated state [72]. E3 ubiquitin ligases, such as Cbl-b, RNF-125 [115], Cullin 1 [116], and TRIM31 [117], have been shown to ubiquitinate NLRP3 and act as negative regulators by mechanisms including but not limited to degradation. Alternatively, Pellino2 has been shown to act as a positive regulator [118]. As such, deubiquitinating enzymes remove K48- and K63-linked polyubiquitin chains, thus stabilizing and promoting NLRP3 expression, as well as inhibiting its degradation [119,120,121]. Additionally, diverging from its role in classical priming, TRAF6 appears to be required for mediating non-transcriptional priming via ubiquitination, as inflammasome activation is unaffected by inhibiting transcription in WT or TRAF6 KO macrophages alike [110]. In response to TLR4/IL-1R signaling, TRAF6 is required for NLRP3 inflammasome activation [110]. Therefore, deubiquitination is a critical step in licensing NLRP3, which is basally in a ubiquitinated state, and bypassing the transcriptionally regulated priming phase for subsequent inflammasome activation [72].

Phosphorylation events are another critical prerequisite for inflammasome activation. c-Jun N-terminal kinase 1 (JNK1) phosphorylated NLRP3 as early as 15 min after LPS dosing and allowed for NLRP3 homotypic interactions/oligomerization, which may be important for the NLRP3 oligomerized cage’s intermediate structure preceding the fully assembled complex [122]. Additionally, this phosphorylation allowed for deubiquitination, a necessary step for activation as described above [122]. Ser194A knock-in mice display an inability for inflammasome activation, revealing the necessity of this PTM in the early phase of priming [122]. Phosphorylation by misshapen-like kinase 1 (MINK1) promoted the priming signal by supporting NLRP3 oligomerization in an ROS-dependent manner [123]. Further, phosphorylation by Casein Kinase 1 alpha 1 (CSNK1A1) during priming allowed for NEK7 binding, and was subsequently dephosphorylated during activation [124]. Inhibition of NLRP3 oligomerization by AKT-mediated Ser5 phosphorylation [125] occurred as a result of electrostatic interference inhibiting homotypic PYD interactions [126]. Additionally, this phosphorylation stabilizes the protein by reducing Trim31-mediated ubiquitination and degradation [125]. Subsequent dephosphorylation by phosphatase 2A (PP2A) permitted activation [126]. Aside from this non-exhaustive list of PTMs on the NLRP3 protein, ASC, IL-1β, and procaspase-1 are also subjected to various PTMs [111,127]. Evidently, these PTMs provide another layer regulating inflammasome assembly.

## 8. Mitochondria as Inflammasome Signaling Hubs

### 8.1. mtROS

Due to the diversity of DAMPs that activate the NLRP3 inflammasome, such as nigericin, ATP, silica, particulate matter, and viral factors [47,128], converging signals have become the focus. ROS have emerged as one of the common signals downstream of these agonists [129]. The literature has characterized ROS as critical activators of the NLRP3 inflammasome [130,131]. However, the role of ROS as inflammasome agonists is still evolving and under debate. Exogenous ROS treatments have appeared to successfully elicit inflammasome activation [132,133]. Further, studies suggest a role for NADPH oxidase (NOXs), a family of ROS-producing membrane enzymes, in contributing to NLRP3 inflammasome activation [134,135]. Despite this, the importance of ROS derived from NOXs has been cast into doubt by NOX inhibition experiments [136,137]. LPS priming and 6 h of Rotenone or Antimycin A (ETC complex I and III inhibitors, respectively) treatment induced NLRP3 inflammasome activation, as measured by caspase-1 cleavage in THP1 and BMDM cells [130]. This is in line with other groups, indicating that mtROS are involved in NLRP3 inflammasome activation [138]. Conversely, the combination of LPS and Rotenone in other studies using the same cell lines was shown to be insufficient for activation [133,139]. Indeed, NLRP3 expression was upregulated with Rotenone alone, and with the presence of ATP, a bona fide activation signal, increased IL-1β, in line with the function of a priming signal [139,140]. This discrepancy between results using Rotenone may be due to varied treatment concentrations and durations. It is known that by reducing mtROS under allergic asthma conditions can lead to a decrease in NF-κB nuclear translocation and NLRP3 activation [141]. However, bypassing the priming requirement by NLRP3 overexpression abrogates the suppressive effects of NAC on inflammasome activation [140]. Additionally, mtROS have been shown to mediate the rapid NLRP3 deubiquitination observed in priming [72]. In summary, mtROS may play an important context-dependent role in the priming phase, and less so in the activation phase. However, it does not appear to be the ultimate trigger of NLRP3 inflammasome as once portrayed [133,139,140,141].

In both C2C12 myotubes and muscle tissue, it has been shown that Angiotensin II induced elevations in mtROS, which then mediated NLRP3 inflammasome activation [59]. Treatment with MitoTEMPO, a mitochondrial antioxidant, blunted this effect in vitro. The reduced mtROS was associated with decreased inflammasome activation and atrophy markers, while protein synthesis markers were elevated [59]. In addition, LPS stimulation itself promotes cellular ROS levels [142,143] and activates the inflammasome, while blunting mtROS levels improved myotube diameter [58]. Interestingly, the mitochondrial irregularities and reduced quantity, the mitochondrial localization of NLRP3, the elevation of mitophagy markers, the reduction in membrane potential, and the muscle atrophy have all been suggested to occur secondary to NLRP3 induction [144]. Mechanistically, it has been postulated that thioredoxin-interacting protein (TXNIP) may bridge the relationship between ROS and NLRP3 activation [145]. Cellular oxidative stress prompts the dissociation of TXNIP from thioredoxin-1 (Trx-1), an antioxidant protein, thereby permitting its binding to and activation of NLRP3 [145].

### 8.2. mtDNA

Accumulating evidence now suggests that mtROS appear to exert their effects on inflammasome activation in a secondary messenger manner by oxidizing mtDNA and promoting its release [131,146,147]. mtDNA possesses several attributes conferring its vulnerability to damage, and therefore is subjected to greater oxidation compared to nuclear DNA [38,148,149]. The proximity of mtDNA to ETCs, lack of protective histone proteins, and insufficient repair mechanisms are all responsible for this [35,36,37,38]. When stress is imposed on mitochondria, the mitochondrial permeability transition pore (mPTP) opens, leading to a cascade of events, including compromised membrane potential, cytochrome c release, cell swelling, and cell death [150,151]. BAX and BAK, members of the Bcl-2 protein family, are implicated in apoptosis, serving to promote outer mitochondrial membrane permeabilization [152]. mtDNA release has also been shown to be mediated by mPTP opening and BAX/BAK channels [153,154]. The application of LPS and ATP treatment, a common protocol for inflammasome activation, decreased membrane potential and promoted apoptosis in macrophages [131]. Downstream of this, it was ox-mtDNA release that was pinpointed as critical for NLRP3 activation. Using cyclosporin A to inhibit the MPTP or Bcl-2 (anti-apoptotic) overexpression blunted inflammasome activation [131]. In fact, 8-OH-dG and mtDNA co-immunoprecipitated with NLRP3 [131,146]. Additionally, activation of the NLRP3 inflammasome was blunted by mtDNA depletion, scavenging of mtROS with Mito-TEMPO, or Cyclosporin A [147]. Interestingly, mtDNA translocation to the cytosol is not just an upstream activator of the NLPR3 inflammasome, as it has also been shown to require NLRP3 [147]. Furthermore, TLR4 activation elicited mtDNA synthesis, which appeared to be oxidized and interact with NLRP3, and was necessary for inflammasome activation, corroborating the previous data [155]. This suggests the ability for priming itself to regulate activation. Additionally, outside of a direct interaction, the DNA sensing cGAS-STING pathway has been proposed as the mechanistic link between DNA and NLRP3 inflammasome activation [156]. Cytosolic DNA is recognized by this pathway by binding to cyclic GMP-AMP (cGAMP) synthase (cGAS), which then induces the synthesis of the secondary messenger cGAMP [157]. cGAMP then binds to STING, which is located on the endoplasmic reticulum [158], but upon activation, it relocates to the Golgi and ultimately elicits [159] NF-κB signaling [160]. Rotenone alone induced mtDNA release into the cytosol via mPTP opening, where cGAS-STING signaling was activated. This ultimately lead to NF-κB nuclear translocation to facilitate the priming step and promote NLRP3 activation [156]. Substantiating this, in C2C12 myotubes, STING appears to be an important regulator of diabetes-related NLRP3 inflammasome activation. In fact, the loss of muscle mass and function and an increase in Murf1 and NLRP3 inflammasome-related markers were all alleviated with STING knock-out [57]. Additionally, STING co-immunoprecipitated with NLRP3, signifying the importance of STING as a regulator of inflammasome activation [57].

### 8.3. Cardiolipin

Outside of ROS and mtDNA, numerous studies have pointed to the role of mitochondria in NLRP3 activation, specifically in their role as organelles for scaffolding. In fact, several factors have been shown to recruit and associate with NLRP3 on the mitochondrial membrane. Unique to the mitochondrial inner membrane is the phospholipid cardiolipin [161]. ROS have been shown to promote cardiolipin translocation to the OMM [162], which can be induced by LPS [163]. Interactions between cardiolipin and NLRP3 [164], as well as caspase-1, have been observed, suggesting a role for cardiolipin acting as an inflammasome activation platform [163]. Notably, the mechanisms behind these results were different, one shown to be ROS-dependent during LPS-mediated priming [163] compared to the ROS-independent mechanism during activation [164]. Inhibiting cardiolipin synthesis via cardiolipin synthase leads to a reduction in NLRP3 inflammasome activity [164,165]. Additionally, cardiolipin has been postulated to regulate NLRP3 activation via its involvement in mitophagy. Cardiolipin translocation to the OMM upon distress, such as elicited by mtROS, promotes binding to LC3b, a mitophagy receptor [162]. However, LPS priming in macrophages promoted the interaction between pro-IL-1α and cardiolipin on the mitochondria, and this study suggested that such interactions may interfere with those of cardiolipin and LC3b, thus impairing mitophagy [166]. Consequently, damaged mitochondria could accumulate, and NLRP3 activation might be heightened. This would provide as additional support for the idea of mitochondria serving as platforms for NLRP3 inflammasome activation. Further work seems required to resolve the role of cardiolipin externalization, mitophagy induction and the triggering of inflammasome activation, particularly in skeletal muscle.

### 8.4. Calcium

Excessive cytosolic calcium (Ca^2+^) levels can activate NLRP3 agonists and induce mitochondrial dysfunction and mtROS production [167]. In fact, LPS priming and ATP stimulation depends on ATP-induced Ca^2+^ mobilization [167]. Additionally, Ca^2+^ promotes the association of NLRP3 and ASC [168]. The role of Ca^2+^ in mediating inflammasome activation in skeletal muscle has not been studied, but given the importance of Ca^2+^ in mediating muscle activation and exercise responses, this is a fruitful area for further research.

### 8.5. Additional Recruitment Mechanisms

**Figure 3:**
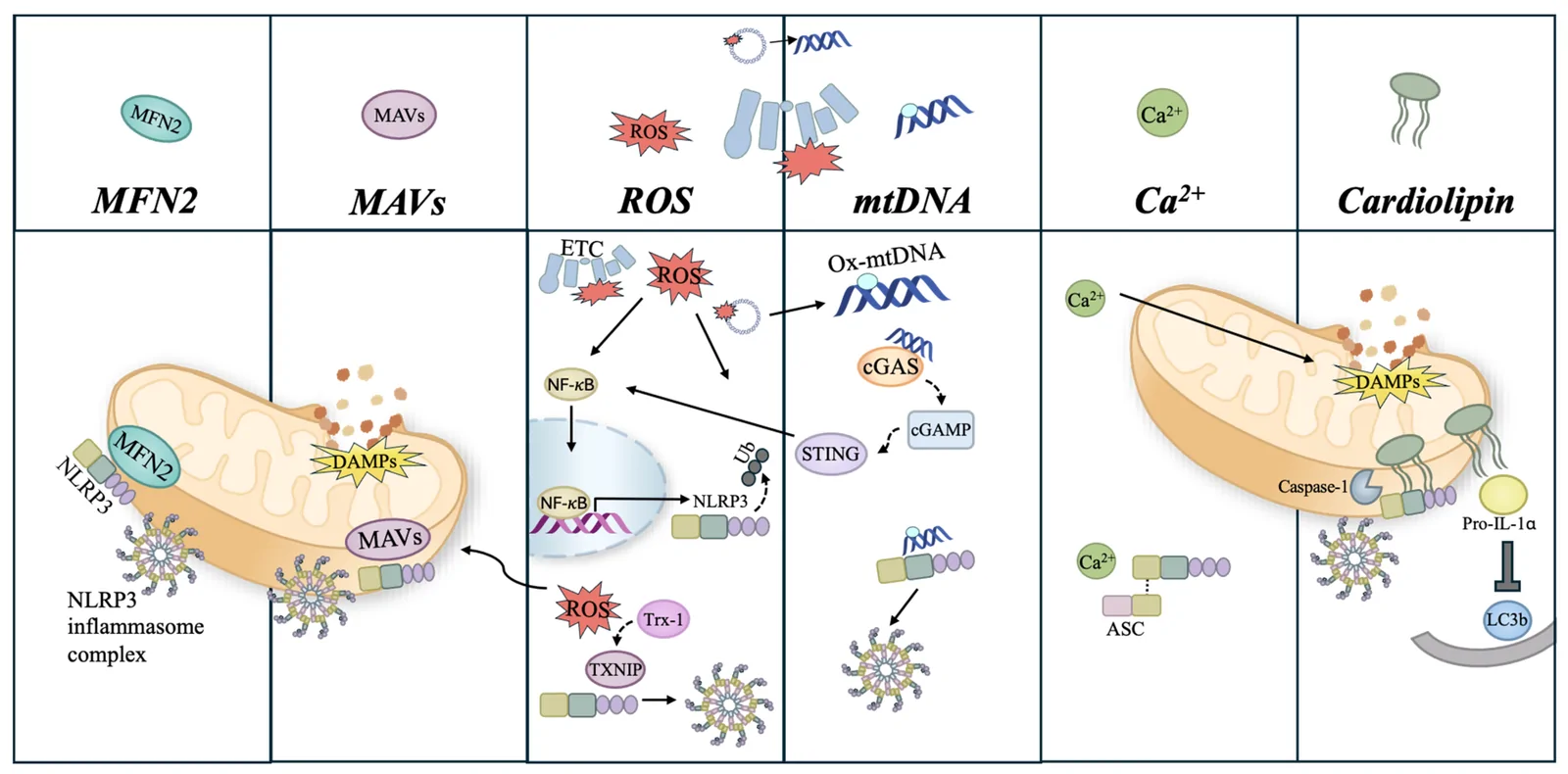
Mitochondria-related NLRP3 inflammasome signaling. MFN2 and MAVs interact with NLRP3 at the mitochondria. ROS influence NLRP3 inflammasome activation by modifying NF-κB signaling and NLRP3 expression, representing the transcriptionally mediated priming step. Additionally, ROS influence rapid priming by mediating deubiquitination of NLRP3. Dissociation of TXNIP from Trx-1 due to ROS promotes NLRP3-TXNIP interaction and NLRP3 inflammasome activation. mtDNA oxidation can result from the presence of elevated ROS, and it can be released into the cytosol where it initiates cGAS-STING signaling to promote NF-κB activation. mtDNA may also directly interact with NLRP3 to facilitate activation. Calcium can augment NLRP3 inflammasome activation by secondary mitochondrial dysfunction. Further, calcium may promote NLRP3-ASC interaction preceding full inflammasome assembly. Cardiolipin directly interacts with NLRP3 and caspase-1, as well as with pro-IL-1α. Pro-IL-1α interactions with cardiolipin compete against LC3b, thereby reducing mitophagy and promoting NLRP3 activation.

Mitofusin-2 (MFN2) has also been demonstrated to interact with NLRP3 under LPS stimulation conditions, and this depends on intact membrane potential, unlike other mechanisms of activation [169]. Additionally, during activation NLRP3 was seen to translocate to the mitochondria mediated by interaction with mitochondrial antiviral signaling proteins (MAVS) to promote NLRP3 activation [170]. MAVS appeared to facilitate NLRP3 oligomerization in an ROS-dependent manner [171]. Indeed, several molecules have been identified for their role in interacting with NLRP3 at the mitochondria. NLRP3 mitochondrial translocation appears to be specifically regulated by the transcription factor STAT3, which interacts with NLRP3 and facilitates trafficking to the mitochondria after the activation signal, thereby promoting activation [172]. Further, STAT3 appears to transcriptionally regulate NLRP3 and IL-1β expression [172]. The various mitochondrial-related signaling pathways described in this section are illustrated in Figure 3.

### 8.6. Autophagy

In order to regulate NLRP3 activation and prevent the deleterious consequences of hyperactive responses, NLRP3, along with the mitochondrial signaling hubs, must be degraded. Congruently, mitophagy/autophagy appears to have an inverse relationship with NLRP3 activation. The inhibition of autophagy leads to an accumulation of dysfunctional mitochondria and NLRP3 inflammasome activation [130]. This relationship appears to be ROS-dependent [147]. PINK1 or PARKIN knockdown leads to elevated NLRP3 activation [173]. Further, this impairment in mitophagy promotes mtDNA release to trigger NLRP3 inflammasome activation [147]. Additionally, the NLRP3 protein contains several KFERQ-like motifs and has been shown to be degraded via chaperone-mediated autophagy (CMA) [174]. This was corroborated in muscle cells, where a receptor involved in the CMA pathway, LAMP2A, was knocked-down and resulted in NLRP3 accumulation, and this process was regulated by the myokine CTRP9 [175]. CTRP9 has been shown to reduce NF-κB DNA binding in cardiomyocytes [176], providing a link between this myokine and NLRP3 priming. NLRP3 accumulation, as due to a blockage of the CMA pathway, resulted in NLRP3 inflammasome activation, atrophy, and senescence [175].

### 8.7. Mitochondrial Localization

The precise spatiotemporal aspects of NLRP3 priming and activation require further characterization. It has been suggested that mitochondrial translocation occurs briefly and early in activation [88]. The function of this has been proposed to be for proximity-related sensitivity to mtDAMPs, thus optimizing the response. NLRP3 temporarily translocated to mitochondria which are in close contact to the Golgi and subsequently translocation to the TGN was observed. This persisted after mitochondrial localization decreased [88]. This corroborates the spatiotemporal movement of NLRP3, in which it translocates from the cytosol to mitochondria and then to the Golgi; however, ASC remains in the cytosol, suggesting that the mitochondria are not the final location for inflammasome assembly and activation [130]. In C2C12 muscle cells, mitochondrial localization of NLRP3 occurred at 72 h, the same time point that IL-1β secretion occurred, suggesting that activation on the mitochondria may occur [58].

## 9. NLRP3 Inflammasome in Skeletal Muscle

NLRP3 activation has been studied in relation to various conditions, such as sepsis [177], masticatory muscle atrophy [144], diabetes [57], ALS [178], and Duchenne muscular dystrophy (DMD) [179]. NLRP3 appears to have protective effects in a model of ALS, as NLRP3 inhibition reduced the survival rate most notably at an earlier time [178]. However, treatment of C2C12 myotubes with IL-1β to recapitulate an environment of sepsis resulted in increased atrophy [177]. This appeared to be associated with upregulated NLRP3 mRNA expression, along with NF-κB promoter activity. Additionally, NLRP3 KO blunted IL-1β secretion and atrophy in a model of sepsis [177]. This is in line with the other in vitro and in vivo literature describing a role for NLRP3 inflammasome in muscle atrophy mediated by atrogenes or impaired protein synthesis [59,144,180]. NLRP3 also appears to contribute to age-related sarcopenia, endurance capacity, and glycolytic capacity [181]. Additionally, the myokine TRIM72 was observed to promote mitophagy and reduce ROS levels, consequently abrogating NLRP3 inflammasome activation in a model of DMD [179].

## 10. Exercise, Muscle, and NLRP3 Inflammasome

**Figure 4:**
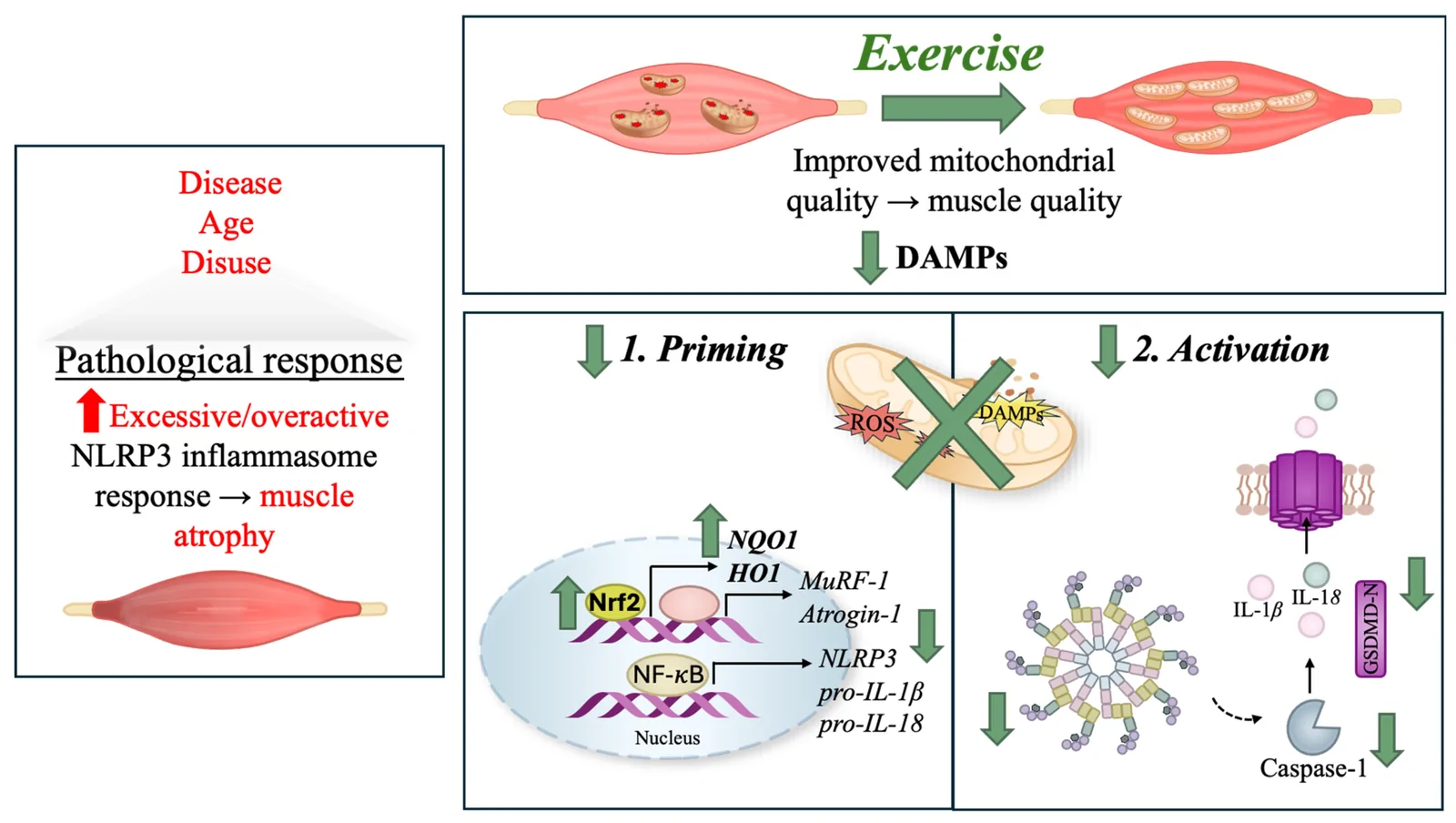
Exercise training reduces NLRP3 inflammasome signaling. In pathological conditions, where NLRP3 inflammasome is upregulated, exercise training promotes a healthy mitochondrial pool by mediating mitochondrial quality control (MQC) pathways. Exercise training elevates Nrf2 signaling, resulting in an increase in antioxidants. These factors contribute to a decline in ROS, which contributes to NLRP3 activation in a variety of mechanisms. Additionally, NF-κB signaling is reduced with exercise, alongside reductions in NLRP3, IL-18, IL-1β, MuRF1, and Atrogin-1. As a result, the NLRP3 inflammasome and its downstream effectors are downregulated and atrophy is blunted.

Although, various drug therapeutics continue to be examined for their ability to alleviate inflammasome-mediated diseases and inflammation [182,183], exercise is a known potent modulator of skeletal muscle health [25]. Endurance training has been extensively characterized in its ability to regulate MQC processes, thereby promoting a healthy mitochondrial pool and reducing ROS [25,30]. Additionally, endurance training combats against muscle atrophy in part by reduced NF-κB signaling [184]. In models of diabetes, 12 weeks of treadmill training attenuated NLRP3, caspase-1, GSDMD, GSDMD-N, IL-1β, and IL-18 [185], while 4 weeks attenuated NLRP3 and IL-18 expression while increasing irisin, a myokine, which appeared to act as negative regulator of this pathway [186]. Atrophy, driven by the upregulation of Atrogin1 and MuRF1 protein expression, was also abrogated with exercise training and NLRP3 inhibition [185]. Additionally, treatment with irisin in C2C12 myotubes mimicked these results [186]. This may be mediated by increased autophagy and reduced oxidative stress, as demonstrated in a microglial cell model of Parkinsons [187]. Meteorin-like (METRNL), another myokine, was demonstrated to be elevated in muscle under 8 weeks of treadmill training and a high-fat diet [188]. In macrophages, suppression of the inflammasome through elevated METRNL appeared to act partially through ERK and p38 MAPK [188]. Congruently, MAPK signaling has been shown to respond to LPS priming [189]. Additionally, 8 weeks of treadmill training rescued elevations in Atrogin-1, MuRF-1, NLRP3, ASC, caspase-1, IL-18, IL-1β induced by a model of chronic kidney disease [190]. This was accompanied by reductions in oxidative stress and autophagy, as well as improvements in mitochondrial content [190]. In an insulin resistance model, 6 weeks of treadmill training lead to upregulations in Nrf-2 and its downstream antioxidants. Additionally, TXNIP, NLRP3, and its downstream markers were downregulated. However, most of these beneficial effects were diminished by Nrf-2 inhibition [191]. Thus, Nrf-2, known for its antioxidant regulation [192] and its downstream targets’ ability to reduce oxidative stress [193,194], appears to be critical in mitigating this inflammatory phenotype. Further, under conditions of aging, NLRP3, caspase-1, and GSDMD protein expression were elevated along with muscle loss, and this effect was alleviated with 6 weeks of voluntary wheel running [56]. Interestingly, STING and BAX appeared to increase with age, while exercise training abrogated this [56]. These data suggest a role for exercise to alleviate an increase in MPTP opening, and thereby reduce the potential escape of mtDNA for subsequent inflammasome activation. Known exercise adaptations, such as improvements in MQC and antioxidant capacity [25], appear to mitigate NLRP3 related pathologies [190,191]. Hence, exercise training may function as a therapeutic, targeting the NLRP3 pathway via mitochondrial health, and should be studied in this context more thoroughly (Figure 4).

## 11. Conclusions

The NLRP3 inflammasome complex is highly implicated in many pathologies, and as such has been well studied in various immune cells. However, as described in this review, muscle cells are capable of propagating their own NLRP3 inflammasome response, although the pathways involved in priming and activation remain to be fully elucidated. Overactivation is often observed in myopathies, resulting in atrophy and muscle weakness. Indeed, this evolutionarily conserved pathway of the innate immune response is critical for host defense and thus complete abrogation is detrimental. Instead, therapeutic measures should target the regulation and protect against overactivation. Research using cell culture models permits the delineation between independent muscle cells and infiltrating immune cells to further our understanding of how muscle contributes to immune signaling. However, this also needs to be extended to pre-clinical models of skeletal muscle in vivo. These studies will be important to continue, so that we can advance our understanding of the mechanisms of NLRP3 activation and the potential therapeutic value of exercise for muscle health.

## Data Availability

No new data were created or analyzed in this study. Data sharing is not applicable to this article.

## Abbreviations

SS	Subsarcolemmal
IMF	Intermyofibrillar
MQC	Mitochondrial quality control
ETC	Electron transport chain
ROS	Reactive oxygen species
mPTP	Mitochondrial permeability transition pore
LPS	Lipopolysaccharide
PAMP	Pathogen-associated molecular pattern
DAMP	Damage-associated molecular pattern
PRR	Pattern recognition receptor
TLR	Toll-like receptor
NLR	Nod-like receptor
NF-κB	Nuclear factor kappa-light-chain-enhancer of activated B cells
IL-6	Interleukin 6
APC	Antigen-presenting cell
MHC	Major histocompatibility complex
Ox-mtDNA	Oxidized mitochondrial DNA
CAPS	Cryopyrin-associated periodic syndrome
FCAS	Familial cold autoinflammatory syndrome
NOMID	Neonatal onset multisystem inflammatory disease
NLRP3	NLR family pyrin domain containing 3
ASC	Apoptosis-associated speck-like protein containing a CARD
LRR	Leucine rich-repeat
PYD	Pyrin domain
FISNA	Fish-specific NACHT-associated domain
NBD	Nucleotide-binding domain
HD1	Helical domain 1
WHD	Winged helix domain
HD2	Helical domain 2
NEK7	NIMA-related kinase 7
CARD	Caspase recruitment domain
IL-1R	Interleukin-1 receptor
MyD88	Myeloid differentiation primary response 88
IRAK	Interleukin 1 receptor-associated kinase
TRAF6	TNF receptor-associated factor 6
TAB	TAK-binding protein
TAK1	Transforming growth factor beta-activated kinase 1
IKK	IκB kinase
IκBα	Nuclear factor of kappa light polypeptide gene enhancer in B-cells inhibitor alpha
TNFR	Tumor necrosis factor receptor
TNF-α	Tumor necrosis factor-alpha
IL-18	Interleukin-18
Cbl-b	Cbl proto-oncogene B
RNF-125	Ring finger protein 125
TRIM31	Tripartite motif containing 31
JNK1	Jun N-terminal kinase 1
MINK1	Misshapen-like kinase 1
CSNK1A1	Casein kinase 1 alpha 1
AKT	Protein kinase B
TGN	Trans-Golgi network
ZDHHC1	zDHHC palmitoyltransferase 1
ZDHHC7	zDHHC palmitoyltransferase 7
dTGN	Dispersed trans-Golgi network
HDAC6	Histone deacetylase 6
MARK4	Microtubule affinity regulating kinase 4
NOX	NADPH oxidase
TXNIP	Thioredoxin-interacting protein
Trx-1	Thioredoxin-1
BAX	Bcl-2-associated X-protein
BAK	Bcl-2 antagonist/killer 1
Bcl-2	B cell lymphoma 2
cGAS	Cyclic GMP-AMP synthase
STING	Stimulator of interferon genes
Murf1	Muscle RING-finger protein-1
MFN2	Mitofusin-2
MAVS	Mitochondrial antiviral signaling protein
STAT3	Signal transducer and activator of transcription 3
PINK1	PTEN-induced kinase 1
CMA	Chaperone-mediated autophagy
LAMP2A	Lysosomal-associated membrane protein 2
CTRP9	Clq-TNF-related protein-9
TRIM72	Tripartite motif-containing 72
METRNL	Meteorin-like
ERK	Extracellular signal-regulated kinase
p38 MAPK	p38 mitogen-activated protein kinase
